# EHMTI-0396. Reappearance of high fever on migraine patients, after individualized homeopathic treatment, is a valuable prognostic factor

**DOI:** 10.1186/1129-2377-15-S1-M7

**Published:** 2014-09-18

**Authors:** S Kivellos, S Skifti, G Vithoulkas

**Affiliations:** 1 Headache Clinic, "G.Gennimatas" Athens General Hospital, Athens, Greece; 2 Academic Dept, International Academy of Classical Homeopathy, Alonnisos, Greece

## Background

Nine years experience of introduction of Homeopathy at Headache Clinic of a major Public Hospital.

## Aim

To reproduce the positive results of a previous study presented in EHTIC 2008.

## Methods

One hundred and twenty migraine patients assigned to receive individualized homeopathic treatment. Additional evaluation by a neurologist was performed at baseline, 6 and 12 months. Primary and secondary measures of migraine severity and impact on quality of life were recorded and analyzed.

## Results

Eighty two patients opted only for homeopathic treatment until the completion of the study, with a baseline HIT-6 score of 67±4. Significant improvement was recorded at 6 months (HIT-6 45±8, P<0.0009 vs baseline, Wilcoxon signed ranks test), further established at 12 months (HIT-6 40.2±7, P<0.0009 vs 6 months). Migraine severity (VAS) decreased by 72% and frequency by 81 % at 12 months (P<0.0001 vs baseline). Observed potential adverse effects were an initial 'aggravation' of migraine symptoms in 69%, recurrence of past medical diseases ,especially infections or upper respiratory with high fever in 62%.

## Conclusion

More positive results and more reappearance of high fever infections appeared in this study after following G.Vithoulkas’ instructions.

Original videos of many patients describing the unexpected reappearance of very high fever, after a long time, will be presented.

No conflict of interest.

